# Allergic contact dermatitis to antiseptic medicated dressing applied on phlebotomy site

**DOI:** 10.4103/0973-6247.67020

**Published:** 2010-07

**Authors:** Hari Krishan Dhawan, Satyam Arora, Suchet Sachdev, R. R. Sharma, Neelam Marwaha

**Affiliations:** *Department of Transfusion Medicine, PGIMER, Chandigarh, India*

## Introduction

Adhesive dressing application to the phlebotomy site is a norm that is usually followed by all blood banks as postdonation care. The purpose is to provide an environment conducive for healing i.e. prevent infection. However whether we need a medicated antiseptic dressing is of doubtful value. The risk of infection at phlebotomy site is estimated to be 1 in 200,000,[1] whereas the reported allergy to medicated antiseptics could reach upto 15%.[2]

## Observation

**Figure 1:**
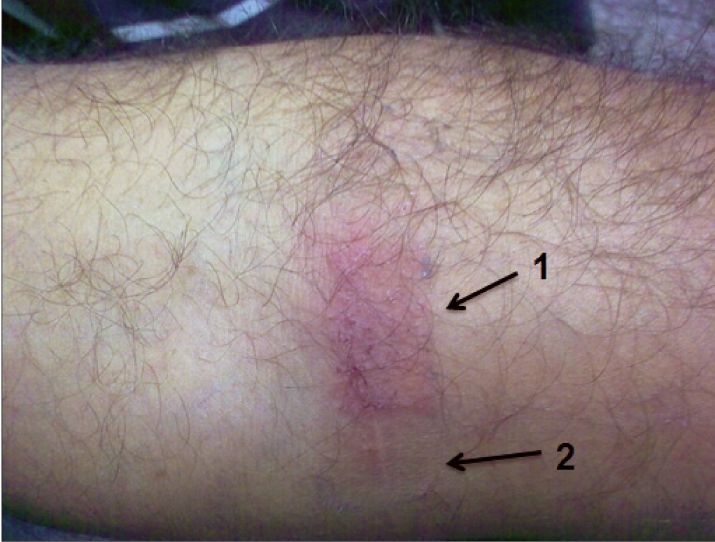
1. The erythematous rash limited to medicated part of dressing. 2. The rash free area which was under the adhesive part of dressing

An erythematous rash developed in one of our first time blood donor, exactly over the area covered under the region of “medicated area” of the adhesive dressing applied post phlebotomy. The donor observed this rash and itching 24 hours after removing the dressing. There was no history of any drug allergy during donor screening [Figure 1].

## Pathophysiology of Clinical Event

The adhesive dressing was medicated with nitrofurazone (0.2%w/w), which is reported to be a known contact medicament-allergen.[3,4,5] The donor was diagnosed to have allergic contact dermatitis (ACD) and was prescribed topical corticosteroids. Resolution of symptoms occurred within 3 days. Sensitivity to both medicament and adhesive part of medicated dressing is reported.[3] Furthermore, breach of skin barrier is known to predispose to such allergy and the contact of even haptens with the exposed proteins results in the formation of complete allergen. The primary presentation of ACD is limited to the typical area of contact similar to the presentation noted in our case in which rash was limited to the medicated part of dressing and area under adhesive part is free from rash. The donor was counseled regarding the allergy to nitrofurazone and to avoid intake as well as contact with this drug in future.

## Preventive Measures

Non-medicated dressings can be considered to avoid such allergies because phlebotomy site is always prepared with an antiseptic and sterile needles are used. This case highlights the need of active donor hemovigilance program so that delayed donor adverse events can be managed appropriately to the satisfaction of the donor.
